# Partial mycoheterotrophy: a common strategy in arbuscular mycorrhizal plants?

**DOI:** 10.1111/nph.71411

**Published:** 2026-07-20

**Authors:** Vincent S. F. T. Merckx, Cas Verbeek, Tomas Figura, Jada Carter, Izai A. B. S. Kikuchi, Franziska E. Zahn, Gerhard Gebauer, Sean W. Graham, Kenji Suetsugu, Deyi Wang

**Affiliations:** ^1^ Naturalis Biodiversity Center 2333 CR Leiden the Netherlands; ^2^ Institute for Biodiversity and Ecosystem Dynamics (IBED), University of Amsterdam 1098 XH Amsterdam the Netherlands; ^3^ Department of Mycorrhizal Symbioses, Institute of Botany Czech Academy of Sciences Lesní 322 25243 Průhonice Czech Republic; ^4^ Department of Botany University of British Columbia Vancouver BC V6T 1Z4 Canada; ^5^ Department of Ecology of Fungi, Bayreuth Center of Ecology and Environmental Research (BayCEER) University of Bayreuth 95440 Bayreuth Germany; ^6^ Laboratory of Isotope Biogeochemistry, Bayreuth Center of Ecology and Environmental Research (BayCEER) University of Bayreuth 95440 Bayreuth Germany; ^7^ Department of Biology Graduate School of Science, Kobe University Kobe Hyogo 657‐8501 Japan; ^8^ Institute for Advanced Research Kobe University 1‐1 Rokkodai, Nada‐ku Kobe Hyogo 657‐8501 Japan; ^9^ Chengdu Institute of Biology, Chinese Academy of Sciences Chengdu 610213 China

**Keywords:** arbuscular mycorrhiza, autotrophy, loss of photosynthesis, mixotrophy, mycoheterotrophy, partial mycoheterotrophy

## Abstract

The vast majority of land plants transfer part of the organic carbon they produce by photosynthesis to arbuscular mycorrhizal (AM) fungi inside their root cells; the fungi in turn help plants to take up nutrients and water from the soil. This carbon can subsequently be acquired from mycorrhizal fungi by rare nonphotosynthetic ‘mycoheterotrophic’ plants that tap into the same fungal network. However, recent findings suggest that carbon uptake from AM fungi may exist among some green plants too. If so, this would qualify them as partial mycoheterotrophs (mixotrophs) rather than as pure autotrophs. Here, we discuss the evolutionary, ecophysiological, morphological, genetic, and environmental evidence for the existence and prevalence of this trait. We conclude that there is strong, albeit indirect, evidence for its existence, although its taxonomic distribution remains to be determined. This knowledge gap currently prevents us from inferring the magnitude of AM partial mycoheterotrophy in terrestrial ecosystems, which in turn severely limits our understanding of its role in plant establishment and survival and in ecosystem structure and function.

## Graphical abstract

Proposed autotrophy to mycoheterotrophy continuum among arbuscular mycorrhizal plants.

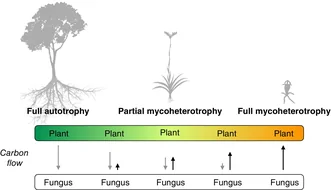

## Mycorrhizal symbiosis

The roots or other underground structures of the vast majority of land plants are associated with mycorrhizal fungi, which help plants to take up water and growth‐limiting nutrients from the soil. Mycorrhizal fungi, in turn, benefit from this interaction by obtaining photosynthetically fixed carbon from the plants. These mycorrhizal interactions mediate soil processes in terrestrial ecosystems globally and are major drivers of ecosystem carbon and nitrogen dynamics (Smith & Read, 2008; Hawkins *et al*., 2023).

Based on the morphology of the interaction and the identity of the interacting partners, five main mycorrhizal types have been described, each with a distinct evolutionary history, anatomy, and ecology; these, in turn, affect plant protection, nutrient acquisition, and belowground carbon and nutrient cycling (van der Heijden *et al*., 2015; Prout *et al*., 2024): (1) the great majority of land plants form arbuscular mycorrhizal (‘AM’) associations with mycorrhizal fungi from Glomeromycotina (a subphylum of Mucoromycota; Spatafora *et al*., 2016). AM fungi form tree‐like (‘arbuscules’) or coil‐like intracellular structures in roots, known as *Arum‐* and *Paris‐*type morphologies, respectively (Gallaud, 1904; Smith & Smith, 1997). These interfaces are the main sites of carbon‐for‐nutrient exchange between plants and their fungal partners; (2) Mucoromycotina fine root endophytes (‘MFRE’) can form intracellular structures in roots that closely resemble AM interactions, in a very wide range of land plants, although they are functionally and evolutionarily distinct from those found in AM systems (Field & Pressel, 2018; Field *et al*., 2019); (3) *c*. 2% of plant species depend on ectomycorrhizal (‘EcM’) associations, which consist of extracellular structures formed by fungi in the phyla Basidiomycota, Ascomycota, and rarely Mucoromycotina (Tedersoo & Smith, 2013; van der Heijden *et al*., 2015). Although few plant species rely on EcM interactions, these interactions are significant for terrestrial ecology because EcM plants cover over 25% of the global vegetation, with many being common tree species, particularly in temperate and subarctic regions (Smith & Read, 2008); (4) fungi forming ericoid mycorrhizas (‘ErM’) that belong to both the Ascomycota and Basidiomycota phyla and form coils mainly inside the roots of Ericaceae, and also in some taxa outside Ericaceae or Ericales (van der Heijden *et al*., 2015; Leopold, 2016); (5) finally, orchid mycorrhizas (‘OrM’) are formed between most orchids and various saprotrophic or EcM fungi that form hyphal coils within the orchid root cortex cells, largely belonging to the Basidiomycota (van der Heijden *et al*., 2015) and to a lesser extent Ascomycota (Dearnaley *et al*., 2012).

## Mycoheterotrophy

**Fig. 1:**
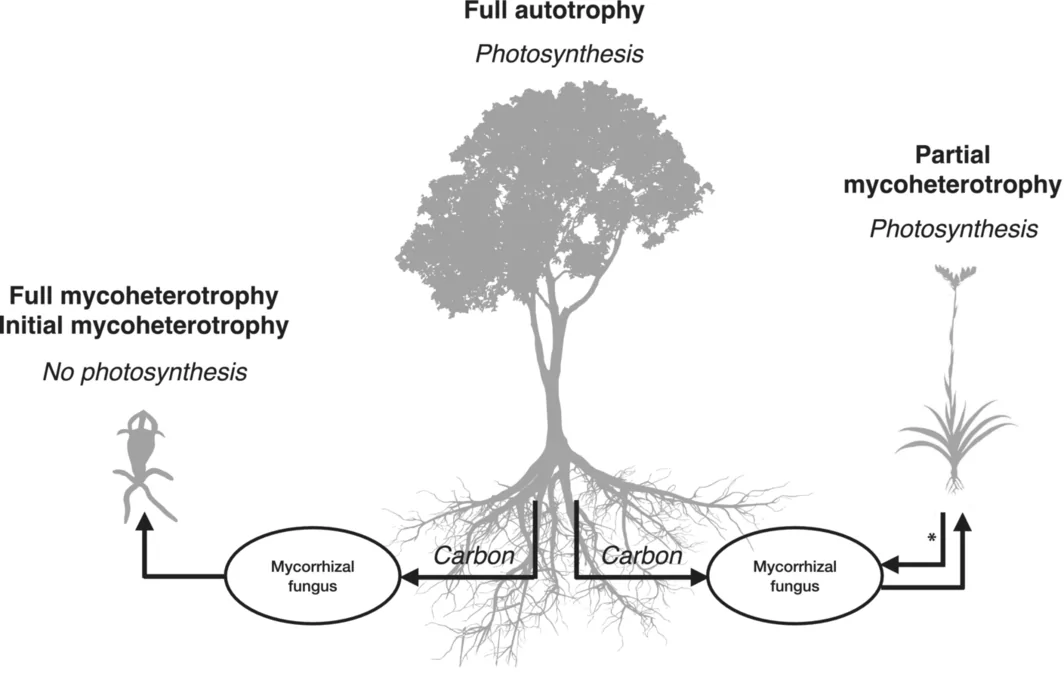
Distinction between autotrophy, mycoheterotrophy, and partial mycoheterotrophy among plants associated with mycorrhizal fungi. Black arrows indicate the flow of carbon, originating from photosynthesis. Asterisk, in partially mycoheterotrophic orchids, there is evidence for bidirectional carbon transfer (Johnson *et al*., 1997; Cameron *et al*., 2006, 2008; Fochi *et al*., 2017), but for arbuscular mycorrhizal mycoheterotrophs, this is not known.

Plants are classically thought to transfer photosynthetically fixed carbon to mycorrhizal fungi, but this paradigm is challenged by the existence of fully mycoheterotrophic plants in which the flow of carbon is reversed: these nonphotosynthetic plants are able to obtain carbon from their root‐associated fungi (Leake, 1994; Merckx, 2013). In most cases, the fungi in the roots of fully mycoheterotrophic plants are AM or EcM fungi that simultaneously associate with roots of surrounding green (i.e. autotrophic) plants, forming a ‘common mycorrhizal network’ (Merckx *et al*., 2024). Hence, the autotrophic mycorrhizal plants are the ultimate source of carbon for fully mycoheterotrophic plants (Fig. 1). However, a few tropical and subtropical mycoheterotrophic orchids are known to obtain carbon from wood‐ and litter‐decaying fungi (Martos *et al*., 2009; Ogura‐Tsujita *et al*., 2009, 2021), and this interaction may be more common than currently assumed (Lee *et al*., 2015).

The fully mycoheterotrophic plants comprise *c*. 580 species of diverse, achlorophyllous plants and are known from angiosperms (eudicots and monocots) and liverworts (Merckx *et al*., 2013a, 2013b, 2013c). They evolved from photosynthetic mycorrhizal ancestors well over 40 times across land plants (Merckx & Freudenstein, 2010; Jacquemyn & Merckx, 2019) and their obligate mycoheterotrophic mode of life has mostly been deduced from the absence of photosynthesis, the absence of a direct (i.e. haustorial) link to any host plant (hence, they are not holoparasites), and dense fungal colonization in their roots or underground structures (Merckx *et al*., 2024). Mycoheterotrophic plants preferentially occur in closed‐canopy forests, supporting the hypothesis that mycoheterotrophy is an adaptation to avoid competition for light in these habitats (Bidartondo *et al*., 2004; Gomes *et al*., 2019b).

However, in some plants, mycoheterotrophy is limited to their early nonphotosynthetic stages of development, while they are photosynthetic during later stages. This phenomenon, known as ‘initial mycoheterotrophy’, is a hallmark of orchids: all *c*. 28 000 species of orchids are thought to be initial mycoheterotrophs; some depend on EcM fungi, but most depend on saprotrophic fungi (Jacquemyn & Merckx, 2019; Selosse *et al*., 2022). Initial mycoheterotrophy on EcM fungi is also known in Ericaceae (Figura *et al*., 2019), whereas initial mycoheterotrophy on AM fungi occurs in several genera of ferns and clubmosses, whose gametophytes are nonphotosynthetic (Merckx *et al*., 2013a, 2013b, 2013c). Because all orchids are initial mycoheterotrophs, and initial mycoheterotrophy is found in multiple clades of land plants, this form of mycoheterotrophy is both taxonomically widespread and present in almost 10% of land plant species.

From a mycological perspective, mycoheterotrophy involves a phylogenetically and physiologically widespread set of fungi (Hynson & Bruns, 2010). For obligate mycorrhizal fungi, the interaction with a mycoheterotrophic plant is often described as the result of parasitism or cheating because of the loss of carbon and the lack of apparent benefits for the fungi; indeed, possible benefits have never been assessed or identified. Nevertheless, the carbon cost imposed by a mycoheterotrophic plant on a fungus may be negligible (Merckx *et al*., 2009).

## Mycorrhizal mixotrophy: a hidden mode of life

Since the advent of mycorrhizal research in the nineteenth century (Frank, 1885), it has been suspected that some achlorophyllous plants obtain carbon from their mycorrhizal fungi (Bidartondo, 2005), but at the beginning of this century, evidence surfaced showing that some adult photosynthetic plants also obtain carbon from EcM fungi. This phenomenon is called ‘partial mycoheterotrophy’ (Gebauer & Meyer, 2003) or (mycorrhizal) ‘mixotrophy’ (Julou *et al*., 2005; Selosse *et al*., 2025) and builds on several lines of indirect evidence. Candidate mixotrophic species are often closely related to fully mycoheterotrophic species (Jacquemyn & Merckx, 2019), and sometimes have vegetative or genomic reductions suggesting a decreased reliance on photosynthesis (Leake, 1994; Julou *et al*., 2005; Girlanda *et al*., 2006; Li *et al*., 2022), often grow in shaded environments (Selosse & Roy, 2009), occasionally include ‘albino’ populations in which plants persist for multiple years despite nonfunctional photosynthesis (Julou *et al*., 2005; Roy *et al*., 2013) and are characterized by carbon (C), nitrogen (N), and/or hydrogen (H) stable isotope signatures intermediate between those of autotrophs and mycoheterotrophs, reflecting the dual photosynthetic and fungal origin of their carbon compounds (Gebauer & Meyer, 2003; Zimmer *et al*., 2007; Hynson *et al*., 2013; Gebauer *et al*., 2016). In particular, evidence from stable isotope signatures has received much attention because it is virtually the only way to search for partial mycoheterotrophy under natural conditions without the need for complicated and artifact‐prone measurements in the field.

Based on this evidence, carbon uptake from EcM fungi has now been established in green orchids and Ericaceae. In addition, carbon pulse‐chase experiments suggest that EcM tree saplings may also be able to obtain small amounts of carbon from their mycorrhizal fungi (e.g. Simard *et al*., 1997; Teste *et al*., 2010; Cahanovitc *et al*., 2022). However, these studies have been criticized on methodological grounds, mostly because they were not able to effectively distinguish carbon transfer through a common mycorrhizal network from transfer through the soil (Karst *et al*., 2023).

For mature EcM partially mycoheterotrophic plant species, the proportional gain of carbon from mycorrhizal fungi has been estimated to range from no carbon gain under some conditions up to 84% (Hynson *et al*., 2013), and the dependence on fungal carbon can differ greatly between populations, seasons, and developmental stages of a plant (Gonneau *et al*., 2014; Schweiger *et al*., 2018; Jacquemyn *et al*., 2021). For EcM partially mycoheterotrophic herbs and seedlings, the amount of carbon taken up is correlated with differences in light availability among plants of the same species, strongly suggesting that plants use this strategy dynamically to compensate for light limitation in shady habitats such as the understory of a dense forest (Preiss *et al*., 2010; Matsuda *et al*., 2012; Gonneau *et al*., 2014). However, a study on partially mycoheterotrophic Ericaceae species at three sites in Estonia with different light availability failed to find differences in carbon uptake (Lallemand *et al*., 2017).

**Fig. 2:**
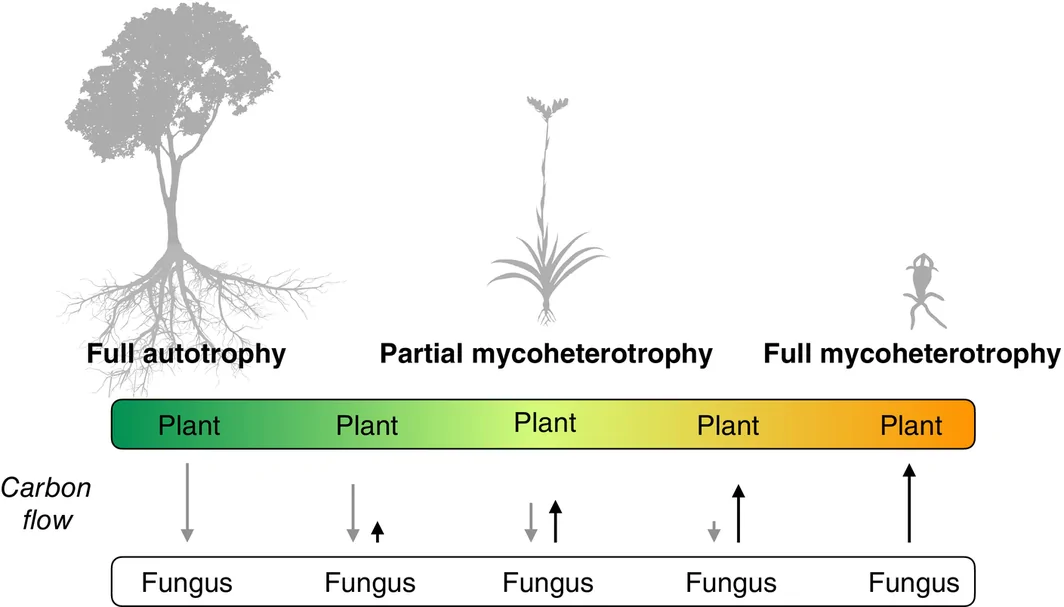
Proposed autotrophy to mycoheterotrophy continuum among mycorrhizal plants, based on Johnson *et al*. (1997).

Phylogenetic reconstructions of Orchidaceae and Ericaceae support the hypothesis that the evolution of mycoheterotrophy is a stepwise process, in which initial mycoheterotrophy or partial mycoheterotrophy is a predisposition for the later evolution of full mycoheterotrophy (Gebauer, 2005; Matsuda *et al*., 2020; Wang *et al*., 2021; Selosse *et al*., 2025). These observations paint a dynamic view of plant–fungal nutritional strategies, in which autotrophy and full mycoheterotrophy represent endpoints of a trophic continuum, with partially mycoheterotrophic states in between. Individual species of orchids and Ericaceae may either occupy a fixed position in this continuum or may shift along the continuum during seasons, phases of plant development, or over environmental gradients (Fig. 2). These patterns are also consistent with a broader evolutionary trajectory across species.

In this context, a clear definition of what constitutes partial mycoheterotrophy is essential. Here, we follow the definition proposed by Merckx (2013): ‘the ability of a plant to obtain carbon simultaneously through autotrophy and mycoheterotrophy’. Importantly, this means that a net carbon transfer to the fungus is still possible (Fig. 2). In orchids, plants showing this pattern have been described as ‘C‐exchangers’ within the ‘Type III mixotrophy’ category (Selosse *et al*., 2025). The reasoning is that in this case, the overall cost of the symbiosis for the plant is reduced, which potentially leads to a competitive advantage in light‐limited habitats. For the plant, gaining even a minute amount of carbon during a particular developmental stage can be a determining factor in the success of plant establishment and development if it outweighs potential costs (Merckx *et al*., 2024). Nevertheless, it is possible that carbon detected in fungus‐to‐plant transfer is associated with the movement of other nutrients that are the primary target of exchange (such as amino acids targeted primarily for nitrogen). If carbon gain by the plant is incidental rather than actively selected for, then this may not strictly speaking qualify as mycoheterotrophy, although this would offer a potential prerequisite for the subsequent evolution of partial mycoheterotrophy. In that sense, any fungus‐to‐plant transfer of carbon warrants investigation in this context.

## The search for AM partial mycoheterotrophy

In view of the existence of AM fully and initially mycoheterotrophic plants and the occurrence of partial mycoheterotrophy in EcM plants, the possibility that green plants can also take up carbon from AM fungi has been raised and tested multiple times over the past few decades. Pulse‐chase experiments using ^14^C (Hirrel & Gerdemann, 1979; Francis & Read, 1984; Grime *et al*., 1987; Lerat *et al*., 2002), ^13^C (Graves *et al*., 1997; Avital *et al*., 2022), or both (Pfeffer *et al*., 2004), as well as variation in the natural abundance of ^13^C, induced by combinations of C_3_ and C_4_ plants (Watkins *et al*., 1996), have all shown that carbon from a ‘donor’ plant reaches the mycelium of AM fungi inside the roots of ‘receiver’ plants, but there is no evidence that it is transferred to the tissue of the latter plants, especially aboveground (Fitter *et al*., 1998; Robinson & Fitter, 1999). In other experiments, labelled carbon was consistently detected in aboveground tissue of AM receiver plants (Grime *et al*., 1987; Lerat *et al*., 2002; Avital *et al*., 2022), but these studies did not show that carbon was transferred by AM fungi rather than via alternative pathways (Bergelson & Crawley, 1988; Pfeffer *et al*., 2004; Karst *et al*., 2023). One particular alternative pathway consists of carbon uptake by roots directly from the soil. Experiments undertaken in hydroponic culture and subsequently in soil have revealed that plant roots can also take up a range of organic compounds from the soil, with subsequent transfer to the shoots (Jones *et al*., 2009). The scale and significance of this process remain unknown (Jones *et al*., 2009). Due to the absence of convincing evidence of AM‐mediated carbon uptake, it has been postulated that in the AM symbiosis, carbon moves unidirectionally from plant to fungus, with fully mycoheterotrophic plants as the only exceptions (Pfeffer *et al*., 2004). As such, this would mean that AM partial mycoheterotrophy is not expected to occur in nature. However, recently, new isotope natural abundance data have once again challenged this paradigm (Cameron & Bolin, 2010; Bolin *et al*., 2017; Giesemann *et al*., 2020a, 2021; Suetsugu *et al*., 2020a, 2020b; Yamato *et al*., 2023; Zahn *et al*., 2024), reinforcing earlier theoretical predictions and observations supporting partial mycoheterotrophy on AM fungi (e.g. Leake, 1994; Jacquemyn & Merckx, 2019).

While from a plant evolutionary perspective, the existence of partial mycoheterotrophy is not unexpected, from a mycorrhizal symbiosis point of view, carbon transfer from a fungus to a plant, potentially without a reward for the fungus, is perhaps counterintuitive. However, it is important to consider that cheating is ubiquitous in mutualisms, as predicted by theory (Bronstein *et al*., 2006), and that the mycorrhizal mutualism is no exception. There are clear indications that both mycoheterotrophic plants and mycorrhizal fungi can behave as cheaters (Walder & van der Heijden, 2015). Experiments indicate that mycorrhizal nutrient exchange dynamics are better understood as community‐wide interactions between multiple players rather than as strict exchanges between individual plants and their symbionts (Durant *et al*., 2023). In addition, our current knowledge from studies of mycoheterotrophy highlights the importance of temporal dynamics of resource exchange in the mycorrhizal symbiosis, which can change in rate and direction over time. While plants can gain carbon from fungi during early developmental stages or particular seasons, they may be autotrophic and thus mutualistic (providing carbon) at other points in time. This may stabilize the symbiosis through selection for net overall fitness benefits for both partners over their lifetimes (Field *et al*., 2015a,b).

## Clues for AM partial mycoheterotrophy

### AM mycoheterotrophy and initial mycoheterotrophy

One of the most straightforward reasons to suspect the occurrence of AM partial mycoheterotrophy is the existence of AM fully mycoheterotrophic plants, which presumably evolved from autotrophs via an intermediate partially mycoheterotrophic state. In angiosperms, there are *c*. 250 species of fully mycoheterotrophic plants in eight families that are associated with AM fungi (Merckx, 2013; Jacquemyn & Merckx, 2019). Phylogenetic reconstructions indicate that they originate from *c*. 16 independent evolutionary shifts from photosynthetic AM plants to fully mycoheterotrophic plants (Merckx *et al*., 2013c) and predominantly occur in tropical and subtropical rainforests, but some species can be found in cloud forests, tropical grasslands, and temperate forests (Merckx *et al*., 2013b). In general, they are considered to be rare, although to some extent, this perception may be due to their ephemeral occurrence and minute habit, which makes them exceptionally hard to spot (Franke, 2007).

Evidence for initial mycoheterotrophy in AM angiosperms is sparse and has so far only been documented for the gentian species *Gentiana zollingeri* (Yamato *et al*., 2021). Outside angiosperms, AM initial mycoheterotrophy is present in several families of ferns and lycophytes (Merckx *et al*., 2013a). In contrast to their photosynthetic sporophytes, the gametophyte phase of these species is achlorophyllous and associated with AM fungi, which presumably act as carbon donors to the free‐living gametophytes (Winther & Friedman, 2008, 2009; Suetsugu *et al*., 2025).

The wide occurrence of AM full mycoheterotrophy throughout the plant tree of life, and the expectation that AM mycoheterotrophy evolves in a stepwise manner – supported by the evolutionary scenarios for EcM and OrM symbioses – suggests that AM partial mycoheterotrophy is likely to exist. The photosynthetic plants most closely related to AM full mycoheterotrophs are prime candidates for AM partial mycoheterotrophy. In this respect, several genera in Burmanniaceae (*Burmannia*) and Gentianaceae (*Exacum*, *Exochaenium*) that contain a mix of green and fully mycoheterotrophic species deserve particular attention (Merckx, 2013). Here, we argue that the characteristics of mycoheterotrophic AM plants and their close relatives will likely be instrumental in the search for cryptic AM partial mycoheterotrophy.

### Natural abundances of stable isotope signatures

Because fully mycoheterotrophic plants are enriched in the heavy stable isotopes of C, N, and H compared to surrounding autotrophs, partially mycoheterotrophic plants are expected to feature enrichments in these isotopes that are intermediate between those of autotrophs and those of full mycoheterotrophs. Using this reasoning, partial mycoheterotrophy has been demonstrated in green Ericaceae and Orchidaceae species (Gebauer & Meyer, 2003; Hynson *et al*., 2013; Gebauer *et al*., 2016).

**Fig. 3:**
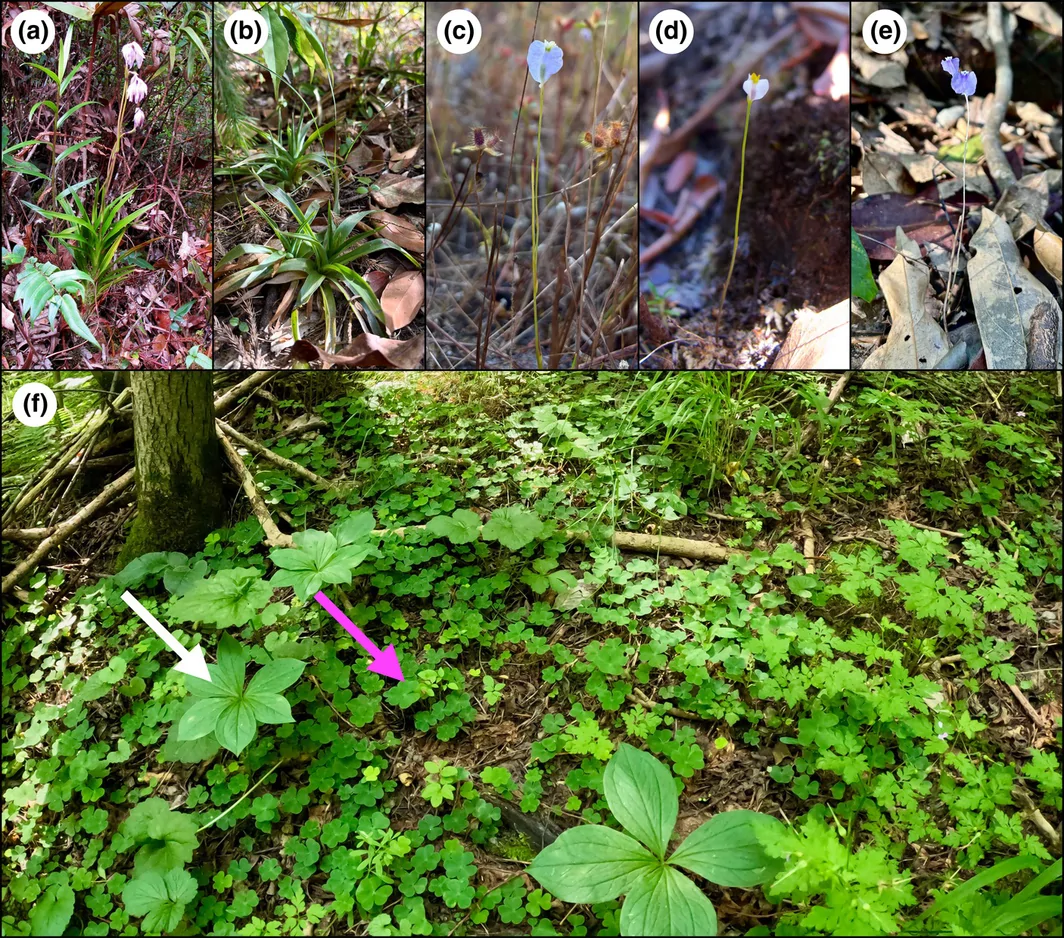
Examples of fully mycoheterotrophic and potentially partially mycoheterotrophic arbuscular mycorrhizal (AM) plants. (a–e) The genus *Burmannia* (Burmanniaceae) includes photosynthetic species with well‐developed vegetative parts, nongreen mycoheterotrophic species with strongly reduced vegetative parts, and species that are positioned between these extremes, suggesting a variable range of abilities to gain carbon from AM fungi across the genus. (a) *Burmannia longifolia* has robust, well‐developed leaves. (b) *Burmannia disticha*, with leaves arranged in a rosette. (c) *Burmannia coelestis* has a slender stem and small rosulate leaves; based on ^13^C enrichment, this species has been identified as a partial mycoheterotroph (Bolin *et al*., 2017). (d) *Burmannia australis* has a slender green stem with few leaves, which are reduced to scales. (e) *Burmannia candida* is achlorophyllous, and the leaves are reduced to scales. (f) A temperate mixed forest in northern Spain, with an understory dominated by *Paris‐*type plants, *Paris quadrifolia* (Melanthiaceae; white arrow) and *Oxalis acetosella* (Oxalidaceae; magenta arrow). Both species have been identified as potentially AM partial mycoheterotrophs by Giesemann *et al*. (2021). All pictures by V. Merckx.

**Fig. 4:**
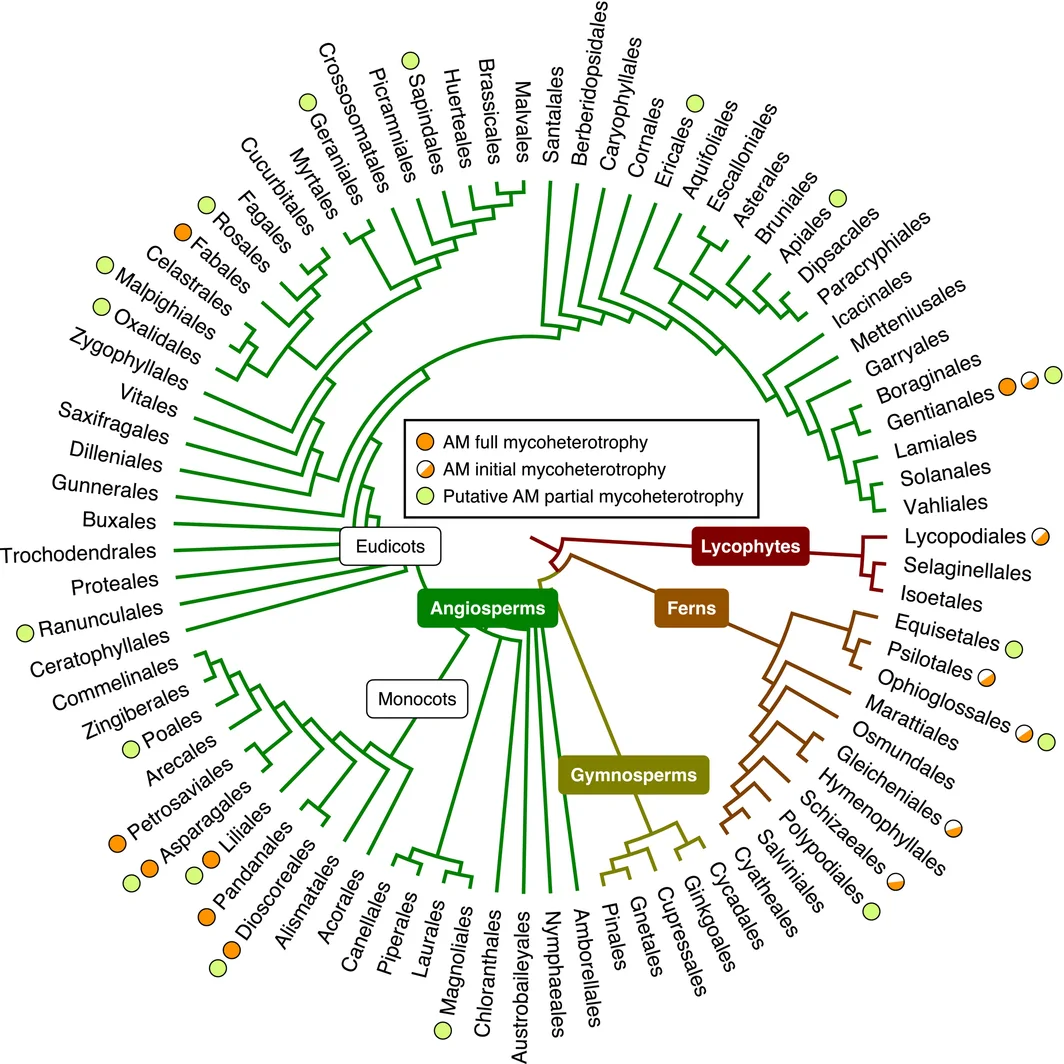
Order‐level occurrence of full mycoheterotrophy and putative partial mycoheterotrophy in arbuscular mycorrhizal (AM) vascular plants, based on currently available data. Phylogenetic relationships following Cole *et al*. (2019, 2021). Occurrence of AM mycoheterotrophy in sporophytes and gametophytes of lycophytes and ferns from Merckx *et al*. (2013a, 2013b, 2013c). Initial mycoheterotrophy in Gentianaceae based on Yamato *et al*. (2021). Occurrence of potential AM mixotrophy based on significant ^13^C enrichments in *Paris*‐type vascular plants reported in Cameron & Bolin (2010), Bolin *et al*. (2017), Giesemann *et al*. (2021), Suetsugu *et al*. (2020a, 2020b), Murata‐Kato *et al*. (2022), Yamato *et al*. (2023), and Zahn *et al*. (2024), and a ^14^C labeling experiment (Lerat *et al*., 2002). Orange circles include species that are AM full mycoheterotrophs; half‐orange circles include species that are AM initial mycoheterotrophs; green circles include putative AM partial mycoheterotrophs.

Whether this framework can be applied to AM systems remained unknown for a long time, as no isotopic signatures of AM mycoheterotrophic plants were available. However, recent work has now revealed that AM mycoheterotrophic plants are indeed enriched in ^13^C, ^15^N, and ^2^H compared to photosynthetic understory plants (Merckx *et al*., 2010; Courty *et al*., 2011; Gomes *et al*., 2020; Zahn *et al*., 2024). This has provided indirect evidence for the identification of partial mycoheterotrophy in plant species that are closely related to AM mycoheterotrophs (Cameron & Bolin, 2010; Bolin *et al*., 2017; Suetsugu *et al*., 2020a; Yamato *et al*., 2023; Fig. 3), and in sporophytes of ferns and horsetails (Giesemann *et al*., 2020b; Suetsugu *et al*., 2020b). Notably, an analysis of stable isotope signatures of understory forest plants in a temperate forest in Germany showed that several species, none of which are closely related to AM full mycoheterotrophs, have isotopic enrichments that would categorize them as AM partial mycoheterotrophs (Giesemann *et al*., 2020a). A follow‐up study using isotopic signatures available in the literature reported that 31 out of 135 investigated AM plants have signatures reminiscent of AM partial mycoheterotrophy (Giesemann *et al*., 2021). An analysis of isotope signatures of understory plants from a deciduous forest in Japan also found that several species are characterized by relatively high ^13^C enrichment (Murata‐Kato *et al*., 2022), further broadening the potential taxonomic range of partial mycoheterotrophy (Fig. 4). However, it cannot be unequivocally excluded that these isotopic enrichments could result from phenomena other than carbon gain from fungi. In temperate forests, for example, seasonal differences in photosynthetic carbon assimilation between species driven by their phenology and by differences in water management may lead to isotopic differences between species growing at the same site (Cernusak *et al*., 2016; Murata‐Kato *et al*., 2022). Plants under drought stress, for example, close their stomata and, as a result, incorporate a higher proportion of ^13^C than neighboring plants that are not experiencing such stress.

**Fig. 5:**
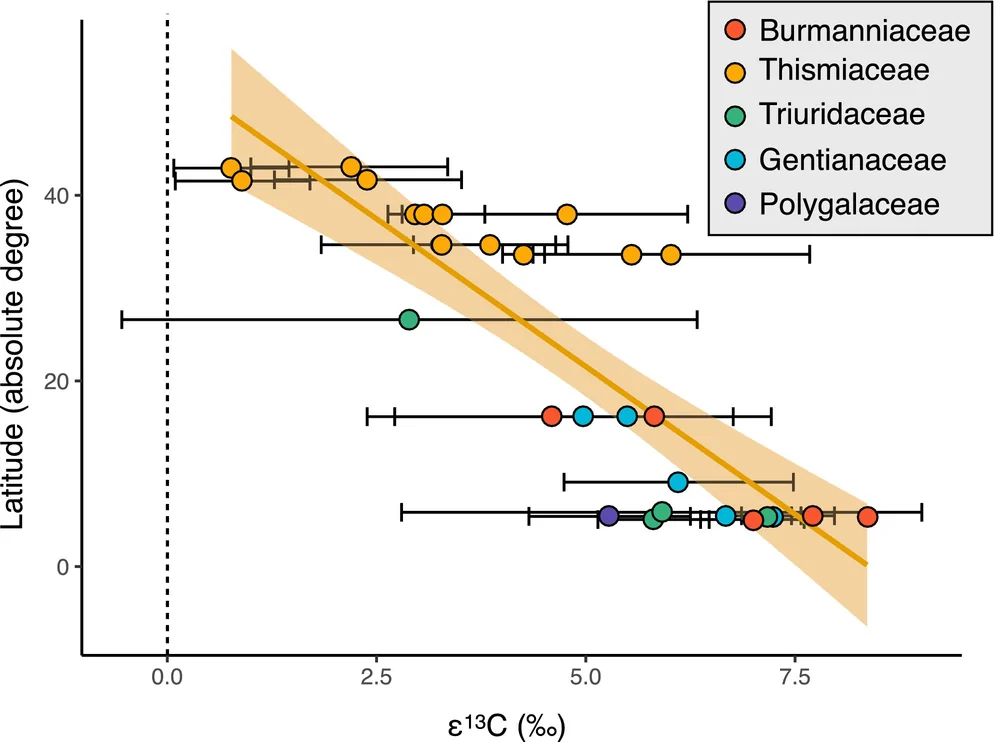
Mean enrichment factors ε^13^C and SD for arbuscular mycorrhizal (AM) full mycoheterotrophs across their latitudinal range in degrees from the equator. Each data point represents the mean enrichment (ε) of several individuals of a fully mycoheterotrophic species at a single site compared to individuals of photosynthetic species at that same site. By considering the mean of the latter at zero for each site, the ^13^C enrichment of mycoheterotrophs can be compared across sites. Data (*n* = 28) from Merckx *et al*. (2010), Courty *et al*. (2011), Gomes *et al*. (2020, 2023), and Zahn *et al*. (2024). A linear regression of the mean ^13^C enrichment factors by latitude is shown in orange (shading indicates the 95% confidence interval); *R* = −0.84, *P* < 0.001.

AM mycoheterotrophs from temperate forests show lower ^13^C enrichment relative to their surrounding green understory plants compared to AM mycoheterotrophs from tropical forests (Fig. 5). AM fungi are enriched in ^13^C compared to their green plant partners (Klink *et al*., 2020), but the degree of this enrichment is further influenced by the ^13^C enrichment of canopy trees (as the main carbon source of AM fungi) relative to understory plants. This difference in ^13^C enrichment may largely result from two factors: (1) the effect of irradiance on ^13^C discrimination in leaves along a vertical profile (Gebauer & Schulze, 1991; Hanba *et al*., 1997); and (2) the fact that understory plants absorb ^13^C‐depleted CO_2_ originating from soil respiration (Murata‐Kato *et al*., 2022). High irradiance enhances photosynthesis, and drier canopy conditions reduce stomatal conductance, which limits equilibration between leaf intercellular and atmospheric ^13^CO_2_ and ^12^CO_2_ concentrations in canopy leaves, thereby reducing isotopic discrimination (Courty *et al*., 2011). This causes ^13^C enrichment in the leaves of canopy trees compared to plants growing in the shaded understory and may explain the ^13^C enrichment in AM full mycoheterotrophs, especially in tropical forests. In addition, compared with temperate forest floors, the ambient CO_2_ in tropical forest floors, where most AM mycoheterotrophic species occur, is likely to be more ^13^C‐depleted due to higher rates of respiratory CO_2_ released from the soil (Murata‐Kato *et al*., 2022). As a result, AM partial mycoheterotrophy based on ^13^C enrichment is more difficult to detect in temperate than in tropical forests, which in turn may lead to an underestimation of partial mycoheterotrophy at higher latitudes. This trend also invalidates the use of enrichment factors derived from tropical AM mycoheterotrophs to represent the mycoheterotrophic endpoint of the autotrophy‐to‐mycoheterotrophy continuum in temperate habitats (Murata‐Kato *et al*., 2022; Fig. 5).

### Mycorrhizal structures, colonization, and interactions

Many candidate partially mycoheterotrophic AM plants have *Paris‐*type mycorrhizal structures, a feature they share with all AM full mycoheterotrophs (Imhof *et al*., 2013; Giesemann *et al*. 2020a, 2021), which differ from the *Arum*‐type, the other major class of AM plants, in their morphology of root colonization. In *Arum*‐type mycorrhizas, fungal hyphae spread between cortical cells and form short‐lived, heavily branched tree‐like symbiotic structures (arbuscules) within cells. In *Paris*‐type mycorrhizas, cortical cells are colonized by intracellular thick coiled hyphae, which occasionally form fine arbuscule‐like ramifications (Smith & Smith, 1997). However, the distinction between the two types is not always easy to make, and a continuum of mycorrhizal structures exists, ranging from *Arum* to *Paris*, depending on both the plant partner and the fungus. In addition, both types are sometimes observed to co‐occur within a single plant (Dickson, 2004; Smith & Read, 2008).

*Paris‐*type mycorrhizas are common in trees and forest herbs (Dickson *et al*., 2007). *Arum*‐type mycorrhizas have been associated with ruderal and cultivated plants, which are usually fast‐growing and do well in sunlight; by contrast, many *Paris*‐type plants seem to be shade‐loving and slow‐growing (Dickson *et al*., 2007). The presence of *Paris‐*type structures in AM mycoheterotrophs, which show a remarkable similarity with the mycorrhizal structures of orchids and mycoheterotrophic Ericaceae, namely coiled hyphae inside plant cells (Imhof, 1999), and in candidate AM mixotrophs, suggests that *Paris‐*type colonization may represent an important predisposition for the evolution of mycoheterotrophy (Perotto & Balestrini, 2024). Although the AM morphological type is known for only *c*. 1000 species, current estimates predict that there are more plant species with *Paris‐*type mycorrhizas than with *Arum‐*type mycorrhizas (Dickson *et al*., 2007). With an estimated 78% (Brundrett & Tedersoo, 2018) of a global total of 374 000 land plant species (Christenhusz & Byng, 2016) capable of forming arbuscular mycorrhizas, this implies that more than 145 000 species form *Paris‐*type arbuscular mycorrhizas.

However, despite the structural similarity of the mycorrhizal interaction between AM full mycoheterotrophs and candidate partial mycoheterotrophs, isotopic enrichments in ^13^C and ^15^N are not exclusive to plants involved in *Paris*‐type mycorrhizas, and not all *Paris*‐type plants are enriched (Giesemann *et al*., 2021; Murata‐Kato *et al*., 2022). In particular, Murata‐Kato *et al*. (2022) found that both *Paris‐* and *Arum‐*type plants show various levels of ^13^C enrichment, and they questioned the use of this approach for the detection of AM partial mycoheterotrophy, although their comparisons may have been influenced by the distances between sampled plants (Zahn *et al*., 2024). Another possible interpretation is that AM partial mycoheterotrophy is not restricted to *Paris‐*type plants. Notably, Murata‐Kato *et al*. (2022) detected a positive correlation between isotopic enrichment and mycorrhizal colonization intensity. Levels of mycorrhizal colonization differ strongly among AM plants, and the roots of AM mycoheterotrophs are among the most densely colonized of all plants, likely due to the high dependence of the plant on the fungus (Leake, 1994). Mycorrhizal colonization is expected to increase with increasing dependence on AM fungi for resources, potentially including carbon, and therefore, high AM colonization is a possible feature of partially mycoheterotrophic AM plants. In addition, in AM mycoheterotrophic plants, the coiled intracellular hyphae in their roots undergo exhaustive degeneration, which is thought to be caused by the plant digesting the fungus (Imhof *et al*., 2013). Such high levels of hyphal degeneration have also been found in understory Colchicaceae and Gentianaceae species by Kusakabe *et al*. (2024), which may be an indication of partial mycoheterotrophy in these taxa. However, hyphal degeneration is not exclusive to AM coils and has also been observed for arbuscules (Alexander *et al*., 1989; Pumplin & Harrison, 2009).

Mycoheterotrophic angiosperms and mycoheterotrophic gametophytes of ferns and lycophytes growing with AM fungi show a general preference for associations with AM fungi from the family Glomeraceae (Winther & Friedman, 2008, 2009; Merckx *et al*., 2012; Perez‐Lamarque *et al*., 2020; but see Suetsugu *et al*., 2025), and as a result, their interactions are more phylogenetically restricted compared to co‐occurring green plants, which tend to grow with a wider phylogenetic range of AM fungi (Gomes *et al*., 2017; Zhao *et al*., 2025). Observations in green and nongreen members of the genus *Burmannia* (Burmanniaceae) suggest that an increased dependence on AM fungi for carbon leads to a gradual loss of interactions with non‐Glomeraceae fungi (Zhao *et al*., 2021). Consequently, partially mycoheterotrophic AM plants are expected to show higher specificity toward Glomeraceae fungi compared to fully autotrophic plants (but see Kusakabe *et al*., 2024). Although fully mycoheterotrophic plants interact with a subset of the available AM fungi, a recent study found that they generally target fungi that are well connected to autotrophic plants (Gomes *et al*., 2022). Targeting well‐connected fungi in the mutualistic network could be a strategy for mycoheterotrophic plants to increase their resistance and resilience during times of environmental perturbation, but it can also help them avoid ‘detection’ by the fungus.

Besides AM fungi, other endophytic fungi, such as dark septate endophytes, may also play a role in plant nutrition and are sometimes associated with isotopic enrichment in AM plants (e.g. Giesemann *et al*., 2020b). It was recently shown that these fungi form common fungal networks between plants, similar to AM fungi (Bock *et al*., 2025), highlighting their potential for carbon transfer. Moreover, MFRE fungi also appear to have many similarities to fungi from Glomeromycotina (Field *et al*., 2015b), which encompasses the AM fungi, but their potential role in carbon transfer needs further research attention.

### Roots and aboveground traits

Fully mycoheterotrophic AM plants show remarkable convergent evolutionary traits, both belowground and aboveground. In contrast to autotrophs, which normally produce extensive roots that ensure adequate absorption of water and nutrients, these functions may be of relatively minor importance in mycoheterotrophic plants due to their dependence on fungal resources (Leake, 1994). Indeed, the subterranean parts of AM full mycoheterotrophs are typically highly modified and characterized by short and thick structures, such as reduced vermiform, coralloid, and tuberous roots, with minimal surface area in contact with soil, and a lack of root hairs. These features are general characteristics of plants that rely strongly on mycorrhizal symbionts (Bergmann *et al*., 2020). Remarkably, some AM fully mycoheterotrophic taxa completely lack these adaptations: for example, some species of Triuridaceae have long filiform roots with root hairs (Maas & Rübsamen, 1986). The root morphology of fully mycoheterotrophic species and closely related green relatives in both *Burmannia* (Burmanniaceae) and *Exochaenium* (Gentianaceae) does not differ substantially, indicating that in these lineages, a complete dependence on fungal carbon does not require extensive root modifications. Therefore, reduced root systems may be indicative of partial mycoheterotrophy, but this may not always be diagnostic, as different lineages may display different sets of traits associated with mycoheterotrophy.

As expected, the shift to full mycoheterotrophy and consequent loss of photosynthesis are accompanied by profound morphological changes in the aboveground traits of mycoheterotrophic plants. These include slender stems with reduced vascular tissues and the reduction of leaves to scales (Leake, 1994; Merckx *et al*., 2013a). A gradient of these aboveground reductions can be observed in the genus *Burmannia*, which includes robust leafy species, species with a basal rosette of leaves, species with slender stems and small basal leaves, plants with reduced leaves, and finally nonphotosynthetic species with only scales (Fig. 3). In AM partial mycoheterotrophs, reduction in these vegetative traits is likely to be manifested only in taxa that are already largely reliant on fungal carbon; therefore, the use of these traits to detect earlier evolutionary stages of AM partial mycoheterotrophy is expected to be limited.

### Seeds, germination, and early development

In Orchidaceae and Ericaceae, initial mycoheterotrophy is hypothesized to be the first step toward a mycoheterotrophic mode of life and is characteristic of all known partially mycoheterotrophic species in these families (Rasmussen *et al*., 2015; Figura *et al*., 2019). Initial mycoheterotrophy in angiosperms is a process in which small ‘dust’ seeds with little reserves rely on carbon from associated fungi for germination and early development, during which they form achlorophyllous protocorms or protocorm‐like structures. This is supported by *in vitro* experiments, in which these seeds cannot germinate without the addition of a carbon source to the growth medium (sucrose or trehalose; Figura *et al*., 2019). In parallel, most AM mycoheterotrophs are known to have very small seeds with little reserves; *Voyria aphylla* (Gentianaceae) even holds the record for the smallest seeds known in plants, *c*. 0.16 × 0.06 mm (Eriksson & Kainulainen, 2011). Among AM plants, dust seeds are not restricted to mycoheterotrophs but are also found in taxa closely related to mycoheterotrophic lineages (e.g. Gentianaceae) and in families with no known mycoheterotrophic or parasitic representatives (Eriksson & Kainulainen, 2011; Baskin & Baskin, 2021). Initial mycoheterotrophy has been demonstrated in Gentianaceae (Yamato *et al*., 2021), but its possible existence elsewhere requires further investigation in other lineages with dust seeds. Decreasing seed size (or more specifically, the mass of the seed reserves) allows for the production of more seeds, but with a trade‐off of having fewer reserves per seed. A dependency on fungal carbon for germination could compensate for these reduced reserves and may eventually facilitate the complete loss of seed reserves (but also see Suetsugu & Tsukaya, 2025). Nevertheless, Zahn *et al*. (2024) did not find a relationship between seed size and ^13^C enrichment among understory plants in a tropical forest.

Similarly, the free‐living gametophytes of some nonseed plant lineages are achlorophyllous and live underground for several years, representing another form of initial mycoheterotrophy. These gametophytes mostly associate with AM fungi and are believed to be entirely mycoheterotrophic (e.g. Winther & Friedman, 2007, Suetsugu *et al*., 2020b; but see Perez‐Lamarque *et al*., 2023). Their mycoheterotrophic mode of life is supported by experiments showing reduced gametophyte growth on medium without sucrose (Whittier, 1972) and inhibition of spore germination by light (Whittier, 1973, 2006). Achlorophyllous gametophytes are known in lycophytes (Lycopodiaceae), leptosporangiate ferns (Schizaeaceae and Gleicheniaceae), and eusporangiate ferns (Ophioglossaceae and Psilotaceae; Merckx *et al*., 2013a). The green sporophytes of ferns and lycophytes with mycoheterotrophic gametophytes are prime candidates for AM partial mycoheterotrophy, as the ability to take up carbon from mycorrhizal fungi is already present in their gametophytes. Stable isotope analyses suggest the presence of AM partial mycoheterotrophy in *Ophioglossum kawamurae* sporophytes, but no significant enrichments were detected in *O. parvum* and *O. vulgatum* (Field *et al*., 2015a, 2015b; Suetsugu *et al*., 2020b); this ability may be context‐dependent within species or lineage‐dependent (Merckx *et al*., 2024).

Initial mycoheterotrophy may not always be a prerequisite for partial mycoheterotrophy in AM plants: stable isotope signatures of *Equisetum* (Equisetales) suggest the presence of AM partial and even full mycoheterotrophy in their sporophytes, whereas their chlorophyllous gametophytes appear to lack initial mycoheterotrophy (Knowlton, 2012; Giesemann *et al*., 2020b). Nevertheless, homosporous ferns or lycophytes with achlorophyllous gametophytes and seed plant species with dust seeds should be prime targets for studies aimed at the discovery of AM partial mycoheterotrophy.

### Genomic features

The plastid and nuclear genomes of mycoheterotrophic plants are characterized by common features related to the loss of photosynthesis and their dependence on a fungal carbon source (Graham *et al*., 2017; Timilsena *et al*., 2023). In particular, the plastid genomes of mycoheterotrophic lineages often undergo a stepwise process of degradation, which may begin before the complete loss of photosynthesis. The plastid‐encoded *ndh* genes associated with the NADH dehydrogenase‐like complex are missing or pseudogenized in all mycoheterotrophic species investigated so far, and this gene set may typically be among the first plastid‐encoded systems to be lost (e.g. Graham *et al*., 2017). Several partially mycoheterotrophic Orchidaceae and Ericaceae species have missing or pseudogenized *ndh* genes, indicating that the loss of NDH function may precede the loss of photosynthesis (Barrett & Davis, 2012; Graham *et al*., 2017; Lallemand *et al*., 2019). Therefore, degradation or absence of *ndh* genes may also be indicative of AM partial mycoheterotrophy, although this is potentially restricted to partially mycoheterotrophic taxa with a strong dependence on fungal carbon, which are already largely dependent on heterotrophic carbon gain. Caution is needed when using the loss of *ndh* genes as an indicator of AM partial mycoheterotrophy because other phenomena may affect *ndh* genes as well (e.g. Ross *et al*., 2016; Fu *et al*., 2023). Nevertheless, most *ndh* genes of the potential AM partially mycoheterotrophic species *Burmannia capitata* (Burmanniaceae), *Obolaria virginica*, and *Bartonia virginica* (Gentianaceae) are either pseudogenes or missing (Lam *et al*., 2018). In addition, losses of *ndh* genes have been reported in green lineages of Gentianaceae (Fu *et al*., 2021), which contain candidate partially mycoheterotrophic AM species (Yamato *et al*., 2021, 2023). Losses of *ndh* genes have also been detected in Schizaeaceae, which include AM taxa with mycoheterotrophic gametophytes (Ke *et al*., 2022).

Mycoheterotrophic plants also show strong reductions in their nuclear genomes compared to photosynthetic species. Based on transcriptome data, Timilsena *et al*. (2023) reported 174 genes missing in all four mycoheterotrophic species studied, including three AM mycoheterotrophs. Most of these missing genes are tied to photosynthesis and so may not be suited to identifying AM partial mycoheterotrophs. However, not all missing genes are associated with photosynthetic activity in model plants or even with plastid function. Their convergent loss in independent mycoheterotrophic lineages could thus be associated with other common traits of mycoheterotrophs and perhaps also partial mycoheterotrophs (Timilsena *et al*., 2023). More detailed transcriptomic evidence and experimental studies are needed to distinguish the roles of these particular genes. Timilsena *et al*. (2023) also observed a reduction in transcript levels in genes for chlorophyll synthesis and photosynthesis in the green species *Burmannia biflora* relative to autotrophic taxa, supporting the potential partially mycoheterotrophic status of this AM species.

Genes involved in fungus‐to‐plant transfer of organic compounds are of particular interest for evaluating partial mycoheterotrophy in AM plants, but unfortunately, we do not yet know which compounds are involved. The nuclear genomes of partially and fully mycoheterotrophic orchid species tend to have multiple copies of trehalase genes compared to their single‐copy status in genomes of autotrophic plants (Li *et al*., 2022). Trehalose is a disaccharide that acts as a storage and transport carbon compound in fungi and is likely to be one of the main carbon sources for these mycoheterotrophic plants. Expression of trehalase genes is also upregulated in partially and fully mycoheterotrophic species of Orchidaceae (Li *et al*., 2022). The investigation of trehalase gene copy number and expression may therefore offer additional clues for the presence of AM partial mycoheterotrophy, although genetic pathways of other organic compounds that are central to the carbon dynamics in the AM symbiosis, lipids in particular (Bago *et al*., 2002; Jiang *et al*., 2017; Keymer *et al*., 2017; Luginbuehl *et al*., 2017), should also be considered.

### Environmental conditions

Most mycoheterotrophic plants are found in the dark understory of forests, and therefore mycoheterotrophy is often seen as an adaptation to low‐light environments. Based on this assumption, the occurrence of partial mycoheterotrophy is expected to increase with decreasing levels of light availability. Indeed, studies using stable isotope natural abundance suggest that light availability plays a major role in the degree of mycoheterotrophy in a few partially mycoheterotrophic orchids and the tribe Pyroleae in Ericaceae (Zimmer *et al*., 2007; Liebel *et al*., 2010; Preiss *et al*., 2010; Hynson *et al*., 2012). Using Ellenberg indicator values, Giesemann *et al*. (2021) found that chlorophyllous plants with *Paris*‐type mycorrhizas were significantly distinguished from chlorophyllous *Arum*‐type species in their preferences for light availability, indicating that *Paris*‐type species occur preferentially in habitats of lower light availability (see also above). For *Paris*‐type ferns and horsetails, but not for seed plants, Ellenberg values show a significant relationship with ^13^C enrichment, which the authors interpreted as proportionally higher mycorrhizal carbon gains under conditions of lower light availability.

However, Zahn *et al*. (2024) showed that potentially partially mycoheterotrophic *Paris*‐type species occupy a broader range of habitats than the shaded understory typically associated with candidate partial mycoheterotrophs. Indeed, some of the identified potential AM partial mycoheterotrophs were the saplings of woody, light‐loving pioneer species (e.g. *Cecropia insignis* and *Croton billbergianus*), which indicates that carbon acquisition through AM fungi may serve different purposes in different green plant species (Zahn *et al*., 2024). Alternatively, this may illustrate the limitations of the use of isotopic signatures for the detection of partial mycoheterotrophy, if isotopic enrichments are caused by other as yet unknown factors. However, photosynthetic relatives of AM mycoheterotrophs in Burmanniaceae, Petrosaviaceae, Gentianaceae, and Polygalaceae often occur in seasonally wet tropical grasslands that are characterized by high levels of irradiance (Maas *et al*., 1986; Kenneally, 1995; Kissling, 2012; Figura *et al*., 2024). In such sunny, wet, and nutrient‐poor habitats, these species tend to co‐occur with carnivorous plants, suggesting that their dependence on AM fungi may be driven by mineral (e.g. N, P) rather than carbon limitation (Givnish *et al*., 1984, 2018). A few studies have reported that AM mycoheterotrophic plants primarily occur in habitats with low mineral nutrient concentrations: Sheldrake *et al*. (2017) reported a phosphorus threshold for the occurrence of AM full mycoheterotrophy in Panama, whereas Gomes *et al*. (2019a) concluded that soil nitrate and the interaction between soil potassium and nitrate concentrations were the best predictors for the occurrence of fully mycoheterotrophic plants in tropical rainforest in Colombia. Based on these observations, we speculate that AM partial mycoheterotrophy is expected to primarily occur in low light and/or low nutrient environments, and that high dependence on AM fungi for soil nutrients may be a prerequisite for the evolution of mycoheterotrophy.

## Conclusions and perspectives

**Fig. 6:**
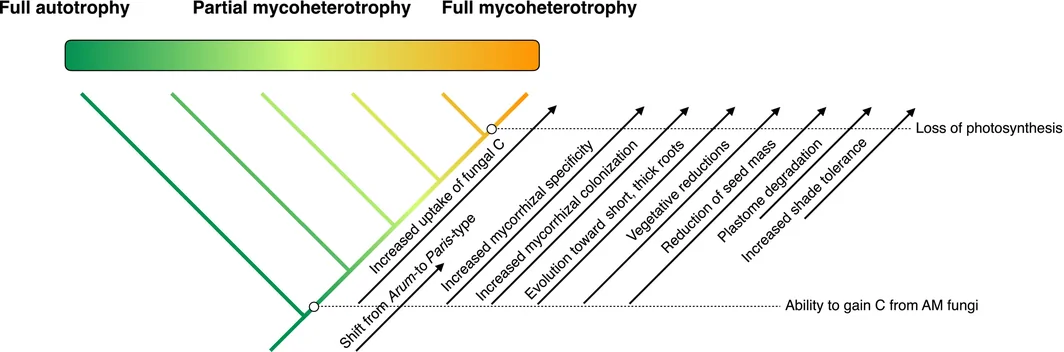
Framework for the evolution of mycoheterotrophy in arbuscular mycorrhizal plants and related characteristics. Arrows indicate evolutionary trends towards increased levels of mycoheterotrophy.

Although there is as yet no direct evidence for the existence of AM partial mycoheterotrophy, observations from various aspects of the biology of AM plants, including their evolutionary relationships, morphology, mycorrhizal interactions, biochemistry, genomics, and plant habitats, reveal a syndrome of plant form and function that strongly suggests that AM partial mycoheterotrophy exists (Fig. 6). For example, *O. virginica* is a green‐leaved species of Gentianaceae, a family that includes fully mycoheterotrophic AM species. It is not closely related to these full mycoheterotrophs but has a suite of features strongly suggesting that it may be partially mycoheterotrophic: its ^13^C enrichment (Cameron & Bolin, 2010), reduced vegetative habit (Leake, 1994), dust seeds (Bouman *et al*., 2002), *ndh* pseudogenes (Lam *et al*., 2018), coralloid roots that are densely colonized by *Paris*‐type AM fungi (Dodds, 2022), and its occurrence in the shaded understory of mature deciduous woods (Dodds, 2022) all point toward this mode of life for this species. Based on a similar assessment, several AM species of angiosperms and ferns, from both forests and open vegetation, potentially qualify as partial mycoheterotrophs (Cameron & Bolin, 2010; Bolin *et al*., 2017; Suetsugu *et al*., 2020a, 2020b, 2025; Ke *et al*., 2022; Yamato *et al*., 2023; Suetsugu, 2025).

Labeled carbon pulse‐chase experiments and physiological observations, such as photosynthetic rates and CO_2_ fixation (e.g. Girlanda *et al*., 2006), are needed to confirm these observations. In addition, an essential outstanding question is whether partial mycoheterotrophy is restricted to a few selected taxa or is instead potentially widespread among AM plants, as suggested by recent stable isotope natural abundance data (Giesemann *et al*., 2021). Because it is not feasible to apply pulse‐chase experiments to a wide range of plant taxa in various ecosystems, the characteristics highlighted here may help to elucidate the prevalence of this mode of life in AM plants. Caution is needed when interpreting individual characteristics in the light of studies on mycoheterotrophy, because no single characteristic provides definite proof of this mode of life. Future research should aim to elucidate which combination of characteristics provides the strongest support for the presence of partial mycoheterotrophy. In addition, other types of measurements, such as photosynthetic rate, may also provide a step forward. However, these methods may fall short of detecting very small carbon gains from AM fungi near the autotrophic endpoint of the autotrophy‐to‐mycoheterotrophy continuum, where partial mycoheterotrophy is likely facultative.

Although there are strong indications for the occurrence of AM partial mycoheterotrophy across a wide range of plant lineages, there are many fundamental unknowns regarding its prevalence and magnitude in plant communities. This limits our understanding of this potentially important, but hitherto overlooked, plant mode of life. Partial mycoheterotrophy not only has potential implications for the germination, establishment, and performance of AM plants across a wide range of species and habitats but also challenges the dogma of a completely unidirectional plant‐to‐fungus carbon flow in the AM symbiosis.

## Competing interests

None declared.

## Author contributions

VSFTM initiated and led the writing of this Viewpoint. CV, TF, JC, IABSK, FEZ, GG, SWG, KS and DW contributed to the writing.

## Disclaimer

The New Phytologist Foundation remains neutral with regard to jurisdictional claims in maps and in any institutional affiliations.
